# Indirect Transfer to Catheterization Laboratory for ST Elevation Myocardial Infarction Is Associated With Mortality Independent of System Delays: Insights From the France-PCI Registry

**DOI:** 10.3389/fcvm.2022.793067

**Published:** 2022-03-11

**Authors:** Farzin Beygui, Vincent Roule, Fabrice Ivanes, Thierry Dechery, Olivier Bizeau, Laurent Roussel, Philippe Dequenne, Marc-Antoine Arnould, Nicolas Combaret, Jean Philippe Collet, Philippe Commeau, Guillaume Cayla, Gilles Montalescot, Hakim Benamer, Pascal Motreff, Denis Angoulvant, Pierre Marcollet, Stephan Chassaing, Katrien Blanchart, René Koning, Grégoire Rangé

**Affiliations:** ^1^Cardiology Department, CHU de Caen, Caen, France; ^2^Cardiology Department, CHU de Tours, Tours, France; ^3^Cardiology Department, Center Hospitalier de Bourges, Bourges, France; ^4^Cardiology Department, CHR de Orléans, Orléans, France; ^5^Cardiology Department, Les Hôpitaux de Chartres, Chartres, France; ^6^Cardiology Department, Clinique Oréliance, Saran, France; ^7^Cardiology Department, Nouvelle clinique Tourangelle, Saint-Cyr-sur-Loire, France; ^8^Cardiology Department, CHU de Clermont-Ferrand, Clermont-Ferrand, France; ^9^Cardiology Department, Groupe hospitalier Pitié-Salpêtrière, Paris, France; ^10^Cardiology Department, Polyclinique les fleurs, Ollioules, France; ^11^Cardiology Department, CHU Nîmes, Nîmes, France; ^12^Cardiology Department, Clinique de la Roseraie, Aubervilliers, France; ^13^Cardiology Department, Clinique Saint Hilaire, Saint Hilaire, France

**Keywords:** ST-elevation myocardial infarction, Pre-hospital, percutaneous coronary intervention, mortality, system delays

## Abstract

**Background:**

First medical contact (FMC)-to-balloon time is associated with outcome of ST-elevation myocardial infarction (STEMI). We assessed the impact on mortality and the determinants of indirect vs. direct transfer to the cardiac catheterization laboratory (CCL).

**Methods:**

We analyzed data from 2,206 STEMI patients consecutively included in a prospective multiregional percutaneous coronary intervention (PCI) registry. The primary endpoint was 1-year mortality. The impact of indirect admission to CCL on mortality was assessed using Cox models adjusted on FMC-to-balloon time and covariables unequally distributed between groups. A multivariable logistic regression model assessed determinants of indirect transfer.

**Results:**

A total of 359 (16.3%) and 1847 (83.7%) were indirectly and directly admitted for PCI. Indirect admission was associated with higher risk features, different FMCs and suboptimal pre-PCI antithrombotic therapy.

At 1-year follow-up, 51 (14.6%) and 137 (7.7%) were dead in the indirect and direct admission groups, respectively (adjusted-HR 1.73; 95% CI 1.22–2.45). The association of indirect admission with mortality was independent of pre-FMC and FMC characteristics. Older age, paramedics- and private physician-FMCs were independent determinants of indirect admission (adjusted-HRs 1.02 per year, 95% CI 1.003–1.03; 5.94, 95% CI 5.94 3.89–9.01; 3.41; 95% CI 1.86–6.2, respectively).

**Conclusions:**

Our study showed that, indirect admission to PCI for STEMI is associated with 1-year mortality independent of FMC to balloon time and should be considered as an indicator of quality of care. Indirect admission is associated with higher-risk features and suboptimal antithrombotic therapy. Older age, paramedics-FMC and self-presentation to a private physician were independently associated with indirect admission. Our study, supports population education especially targeting elderly, more adequately dispatched FMC and improved pre-CCL management.

## Introduction

Primary percutaneous coronary intervention (PCI) is the first-line treatment for ST-elevation myocardial infarction (STEMI) (1). System delays from the first medical contact (FMC) to cardiac catheterization laboratory (CCL) are associated with poor outcome (2–4) and should be reduced to the minimum (1). However, despite improved door to balloon times, within the past decades, rates of mortality seem to be unchanged in the setting of STEMI (5) and further investigations are needed to identify other pre-CCL correlates of outcome and to develop new strategies to improve outcome.

A direct admission to CCL by Pre-hospital emergency medical services (EMS) is recommended in order to reduce system delays (1, 6). Indirect admission to the CCL and the number of contacts, after the FMC, are associated with longer system delays and total ischemic time but their impact on mortality, independent of system delays remains controversial (7, 8).

The objective of our study was to investigate whether indirect admission to CCL impacted mortality independent of FMC to balloon time and other identified co-variables in patients enrolled in a STEMI networks within 24 h following symptom onset and admitted to CCL for primary PCI. We also aimed to assess the determinants of indirect admission.

## Materials and Methods

### Study Design and Population

The France PCI registry is an open, ongoing prospective multicenter registry (clinicaltrials.gov#NCT02778724) including consecutive patients admitted to CCLs for coronary interventions in different French regions as described previously (9, 10). In the setting of STEMI, pre-CCL management, timelines, reperfusion strategies, antithrombotic therapy and intervention characteristics are recorded in patients' medical files and electronically transferred to the registry. In-hospital and 1-year outcomes, derived from source files and/or physical or phone contacts are recorded. The quality of the registry is guaranteed by systematic audits by study coordinators independent of each center.

The current retrospective analysis focused on STEMI patients included within 24 h after symptom onset, undergoing primary PCI between January 1st 2014 and December 31st 2016, excluding patients with out-of-hospital cardiac arrest prior to STEMI, lytic therapy, and no attempt to PCI.

FMC was defined by the first person competent to obtain and interpret the ECG and, to provide initial intervention (1). The standard of care for the management of suspected STEMI in France is the physician-on board ambulance sent to the scene. However, paramedics-on board ambulances and self-presentation to an emergency department (ED) or to a private medical doctor (MD) are other potential pathways. “Door” was defined by the time of admission in the PCI-capable hospital and “balloon” was the time of coronary lesion crossing by the first PCI guidewire or balloon. Successful PCI was defined by a visually assessed coronary diameter stenosis <30% and a thrombolysis in myocardial infarction coronary flow 3. For the purpose of the current study we defined two groups based on the pathway from FMC to CCL: direct admission (guideline-recommended pathway without admission in any other medical facility prior to CCL; e.g., transfer from scene or an ED or ward to CCL) and indirect admission (admission to another medical facility prior to CCL; e.g., private MD or EMS to non-PCI facility then to CCL).

### Outcomes

The primary outcome was mortality of any cause at 1-year follow-up. Secondary outcomes included hospital discharge and 1-year mortality, cardiovascular mortality (death related to cardiovascular causes or sudden death), major bleeding defined by the bleeding academic research consortium (BARC) classification ≥ 3, Non-fatal myocardial infarction, defined by types 1, 4 and 5 based on the universal definition (11), stroke, definite/probable stent thrombosis based on the academic research consortium criteria (12) and unplanned coronary revascularization.

### Statistical Analysis

Baseline data are shown as *n* (%) and mean ± standard deviation and compared between groups using the χ2 test and the Student's *t* test for categorical and continuous variables, respectively. The primary outcome and 1-year cardiovascular mortality were compared between the two groups using Kaplan-Meier curves and the log-rank test. The association between the group and the latter outcomes was assessed using un-adjusted and adjusted on FMC to balloon time (primary analysis) cox regression models with calculation of Hazard ratios (HR) and 95% Confidence intervals (CI). Other 1-year outcomes were assessed by the unadjusted Cox analyses.

Supplementary analyses were performed by adjusting the Cox models on covariables unequally distributed between the two groups with a *p* value < 0.1 in order to assess: 1. the impact of patient characteristics at the time of FMC; 2. The impact of pre-CCL management; 3. The concomitant impact of all latter variables 4. The impact of all latter variables and in-hospital variables.

Sensitivity analyses were conducted using the number of pre-CCL contacts as a continuous variable (1, 2, ≥3) instead of direct vs. indirect admission and total ischemic (symptom-to-balloon) time in replacement of FMC to balloon time in the latter models.

The associations between the groups and in-hospital outcomes were assessed by unadjusted and, in case of significant associations, by adjusted on FMC-to-balloon time logistic regression models with calculation of odds ratios (OR) and 95% CI.

The determinants of indirect admission to CCL were explored using a multivariable logistic regression model including all patient characteristics at the time of FMC unequally distributed between groups (*p* < 0.1) as well as EMS number call, type of FMC and FMC-to-balloon time. Finally, in a final exploratory analysis, we compared data between FMC groups with the MD-EMS group considered as the reference group.

All tests were two sided and a *p* value < 0.05 was considered as statistically significant.

## Results

### Population Characteristics

A total of 2,760 consecutive patients were admitted during the analysis period among whom 2,206 fulfilled the inclusion criteria.

**Figure 1:**
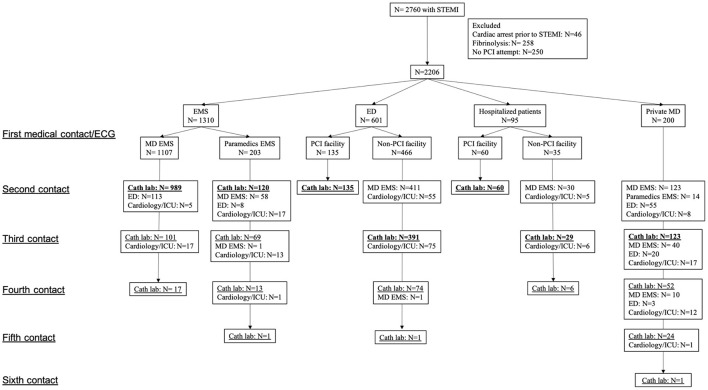
Flow chart and details of first medical contact to cardiac catheterization laboratory pathways. Direct admission to catheterization laboratory appears in bold text. STEMI, ST elevation myocardial infarction; PCI, percutaneous coronary intervention; EMS, emergency medical services; ED, emergency department; MD, medical doctor; ICU, intensive care unit; Cath lab, catheterization laboratory.

Figure 1 depicts the flow chart and pathways from the FMC to CCL. The FMC was an EMS team in 1,310 (59.4%), an ED in 601 (27.2%), a private MD in 200 (9.1%) and a physician in a hospital where patients were already admitted at the time of STEMI in 95 (4.3%) cases. A total of 359 (16.3%) and 1,847 (83.7%) were indirectly and directly admitted to CCL, respectively. The national EMS number was called directly by 1,245 (56%) patients for whom MD- or paramedics-EMS team were sent on scene in 1,090 (88%) and 110 (9%), respectively while others were directed to their MD (0.5%) or an ED (2.5%). Among the 961 (44%) patients who did not call directly the national EMS number, 95 (10%) were already hospitalized for another condition, 601 (62.5%) self-presented to an ED, 200 (20.8%) self-presented to their MD and 65 (6.7%) were referred to the EMS by their MD after a phone call.

Table 1 depicts patient characteristics based on direct vs. indirect admission to CCL. Compared to directly admitted patients, those indirectly transferred were significantly older (*p* = 0.0006) with higher rates of female gender (*p* = 0.01), Killip class ≥1 (*p* = 0.008), diabetes (*p* = 0.01), hypertension (*p* = 0.02) and, lower rates of past history of MI (*p* = 0.002) and PCI (*p* = 0.0009). FMC was less often an MD-EMS (*p* < 0.0001), an ED (*p* = 0.003) or a ward MD (*p* = 0.007) and more often a paramedics-EMS (*p* < 0.0001) or a private MD (*p* < 0.0001) in those indirectly transferred.

**Table 1:** Patient and procedure characteristics.

	**Global population**	**Indirect admission**	**Direct admission**	***P* value**
	***N* = 2,206**	***N* = 359**	***N* = 1,847**	
Age, years	62.66 ± 13.91	65.07 ± 14.68	62.19 ± 13.71	0.0006
Gender, female	547 (25)	108 (30.1)	439 (23.8)	0.01
BMI, Kg.m^−2^	26.63 ± 4.37	26.87 ± 4.81	26.59 ± 4.27	0.3
Killip class > 1	335 (15)	71 (19.8)	264 (14.3)	0.008
Cradiogenic shock	82 (3.7)	19 (5.3)	64 (3.4)	0.09
**Past history**				
Diabetes	295 (13)	63 (17.5)	232 (12.6)	0.01
Hyperlipemia	814 (37)	140 (39)	674 (36.5)	0.4
Current smoking	838 (38)	122 (34)	716 (38.8)	0.09
Hypertension	882 (40)	163 (45.4)	719 (38.9)	0.02
PCI	277 (13)	26 (7.2)	251 (13.6)	0.0009
Myocardial infarction	177 (8)	14 (3.9)	163 (8.8)	0.002
Stroke	53 (2)	12 (3.3)	41 (2.2)	0.2
PAD	72 (3)	11 (3.1)	61 (3.3)	0.8
**Prehospital data**				
EMS number call by patient	1245 (56)	166 (46.2)	1079 (58.4)	<0.0001
FMC				
EMS	1310 (59)	201 (56)	1109 (60)	0.2
MD-EMS	1107 (50)	118 (32.9)	989 (53.5)	<0.0001
Paramedics-EMS	203 (9)	83 (23.1)	120 (6.5)	<0.0001
ED	601 (27)	75 (20.9)	526 (28.5)	0.003
PCI-facility	135 (6)	0 (0)	135 (7.3)	<0.0001
Non-PCI-facility	466 (21)	75 (20.9)	391 (21.2)	0.9
Private MD	200 (9)	77 (21.4)	123 (6.7)	<0.0001
Hospitalized patients	95 (4)	6 (1.7)	89 (4.8)	0.007
PCI-facility	60 (3)	0 (0)	60 (3.2)	<0.0001
Non-PCI-facility	35 (1.6)	6 (1.7)	29 (1.6)	0.9
**Times, min**				
Symptom to FMC	175 ± 217	228 ± 238	165 ± 212	<0.0001
FMC to door	93 ± 96	176 ± 158	76 ± 67	<0.0001
Door to balloon	56 ± 131	107 ± 262	46 ± 82	<0.0001
FMC to balloon	149 ± 166	283 ± 315	123 ± 98	<0.0001
FMC to balloon <120'	1222 (55)	55 (15.3)	1167 (63.2)	<0.0001
FMC to balloon <90'	745 (34)	20 (5.6)	725 (39.3)	<0.0001
Total ischemic time	324 ± 284	511 ± 397	287 ±240	<0.0001
**Pre-catheterization medication**				
Aspirin	2098 (95)	344 (95.8)	1754 (95)	0.5
P2Y12 inhibitors	2030 (92)	332 (92.5)	1698 (91.9)	0.7
Clopidogrel	251 (11)	60 (16.5)	191 (10.3)	0.0005
Prasugrel	135 (6)	17 (4.5)	118 (6.4)	0.2
Ticagrelor	1644 (75)	255 (71)	1389 (75.2)	0.1
IV anticoagulation	1968 (89)	306 (85.2)	1662 (90)	0.008
Enoxaparin	845 (38)	146 (40.7)	699 (37.8)	0.3
UFH	1098 (50)	150 (41.8)	948 (51.3)	0.0009
Bivalirudin	22 (1)	5 (1.4)	17 (0.9)	0.4
**In-hospital data**				
Per-procedure medication				
Aspirin	382 (17)	70 (19.5)	312 (16.9)	0.2
P2Y12 inhibitor	174 (8)	25 (7)	149 (8.1)	0.5
Clopidogrel	24 (1)	4 (1.1)	20 (1.1)	0.96
Prasugrel	11 (0)	3 (0.8)	8 (0.4)	0.3
Ticagrelor	139 (6)	18 (5)	121 (6.6)	0.3
2B3A inhibitor	830 (38)	103 (28.7)	727 (39.4)	0.0001
IV anticogulation	1141 (52)	208 (57.9)	933 (50.5)	0.01
Enoxaparine	19 (1)	3 (0.8)	16 (0.9)	0.95
UFH	1118 (51)	203 (56.5)	915 (49.5)	0.02
Bivalirudin	10 (0)	3 (0.8)	7 (0.4)	0.2
Transradial	1847 (83)	315 (88)	1690 (92)	0.02
Left main disease	4 (0)	1 (0.3)	3 (0.2)	0.6
Single vessel disease	1012 (46)	150 (41.8)	862 (46.7)	0.09
Single vessel PCI	2105 (95)	340 (95)	1765 (96)	0.5
Stents per patient	1.19 ± 0.8	1.24 ± 0.85	1.19 ± 0.79	0.3
Successful PCI	2151 (98)	342 (95.3)	1809 (97.9)	0.003
Medication at discharge	*N* = 2092	*N* = 327	*N* = 1765	
Aspirin^a^	2066 (99)	324 (99)	1742 (99)	0.7
P2Y12 inhibitor	2062 (98.6)	320 (97.9)	1742 (98.7)	0.2
Clopidogrel	282 (13)	61 (18.7)	221 (12.5)	0.003
Prasugrel	181 (9)	23 (7)	158 (9)	0.3
Ticagrelor	1599 (76)	236 (72.2)	1363 (77.2)	0.048
Medication at 1 year^b^	*N* = 1922	*N* = 277	*N* = 1564	
Aspirin	1841 (96)	277 (96)	1564 (96)	1
P2Y12 inhibitor	1006 (53)	156 (54)	850 (52)	0.6
Clopidogrel	255 (13)	44 (15)	211 (13)	0.3
Prasugrel	73 (4)	10 (3.5)	63 (4)	0.7
Ticagrelor	678 (35)	102 (35)	576 (35)	1

a *10 patients with missing data*.

b *281 and 290 patients with missing data at 1 year with respect to aspirin and P2Y12 inhibitor treatment*.

*FMC, first medical contact; BMI, body mass index; ED, emergency department; PCI, percutaneous coronary intervention, PAD, peripheral arterial disease; EMS, emergency medical services; MD, medical doctor; IV, intravenous, UFH, unfractionated heparin*.

The rate of patients with a FMC to balloon time < 120 min was >3 times lower (*p* < 0.0001) and all delays were longer (*p* < 0.0001) in patients indirectly transferred.

Between FMC and CCL, patients indirectly transferred to the hospital were less likely to receive intravenous anticoagulation (*p* = 0.01) and received more frequently clopidogrel (*p* = 0.002) as compared to newer P2Y12 inhibitors.

During the procedure, 2B3A inhibitors and supplementary doses of intravenous anticoagulation were more frequently used in those indirectly transferred (*p* < 0.0001 and *p* = 0.01, respectively). Angiographic findings and PCI characteristics were similar between the groups but the rates of transradial approach and successful PCI were lower in those indirectly transferred (*p* = 0.02 and *p* = 0.003).

At hospital discharge, patients indirectly transferred were more frequently on clopidogrel (*p* = 0.003) and less frequently on ticagrelor (*p* < 0.05).

### Follow-Up and Outcomes

Outcome data (Table 2) were complete for all patients at hospital discharge and 2,124 (96.3%) patients at 1-year follow-up.

**Table 2:** Outcomes and their association with indirect vs. direct transfer.

	**All**	**Indirect transfer**	**Direct transfer**	**Un-adjusted**		**Adjusted on FMC-to-balloon time**	
**In-hospital outcomes**	***N** **=** **2,206***	***N** **=** **359***	***N** **=** **1,847***	**OR (95% CI)**	**Wald** ***p***	**OR (95% CI)**	**Wald** ***p***
Death	114 (5.2)	32 (8.9)	82 (4.4)	2.11 (1.36–3.19)	0.0006	1.84 (1.16–2.85)	0.008
Cardiovascular death	86 (3.9)	26 (7.2)	60 (3.2)	2.33 (1.43–3.70)	0.0005	2.08 (1.25–3.36)	0.004
Myocardial infarction	25 (1)	7 (1.9)	18 (1)	2.02 (0.78–4.68)	0.1		
Stroke	10 (0)	1 (0.3)	9 (0.5)	0.57 (0.03–3.05)	0.6		
Unplanned revascularization	34 (2)	8 (2.2)	26 (1.4)	1.60 (0.67–3.40)	0.3		
Stent thrombosis	26 (1.1)	4 (1.1)	22 (1.2)	0.94 (0.27–2.60)	0.9		
Major bleeding	48 (2.2)	10 (2.8)	38 (2.1)	1.36 (0.64–2.66)	0.4		
**1-*****year*** **outcomes**	***N** **=** **2,124***	***N** **=** **347***	***N** **=** **1,777***	**HR (95% CI)**	**Score** ***p***	**HR (95% CI)**	**Score** ***p***
Death	188 (9)	51 (14.6)	137 (7.7)	2.02 (1.46–2.78)	<0.0001	1.73 (1.22–2.45)	0.002
Cardiovascular death	118 (5.6)	35 (10)	83 (4.7)	2.28 (1.53–3.38)	<0.0001	2.01 (1.31–3.07)	0.001
Myocardial infarction	52 (2.4)	11 (3.2)	41 (2.3)	1.44 (0.74–2.80)	0.3		
Stroke	20 (0.9)	2 (0.6)	18 (1)	0.59 (0.14–2.56)	0.5		
Unplanned revascularization	128 (6)	19 (5.4)	109 (6.1)	0.95 (0.58–1.54)	0.8		
Stent thrombosis	39 (1.8)	6 (1.7)	33 (1.9)	0.99 (0.41–2.35)	1		
Major bleeding	79 (3.7)	13 (3.7)	66 (3.7)	1.06 (0.94–1.93)	0.8		

*FMC, first medical contact*.

At hospital discharge 114 (5.2%) patients were reported dead, 32 (8.9%) in the indirect admission and 82 (4.4%) in the direct admission groups, respectively (adjusted-OR 1.84, 95% CI 1.16–2.85, *p* = 0.008). In-hospital cardiovascular death occurred in 26 (7.2%) and 60 (3.2%) patients in the indirect and direct admission groups, respectively (adjusted OR 2.08, 95% CI 1.25–3.36, *p* = 0.004). Other end points were similarly distributed between the two groups.

**Figure 2:**
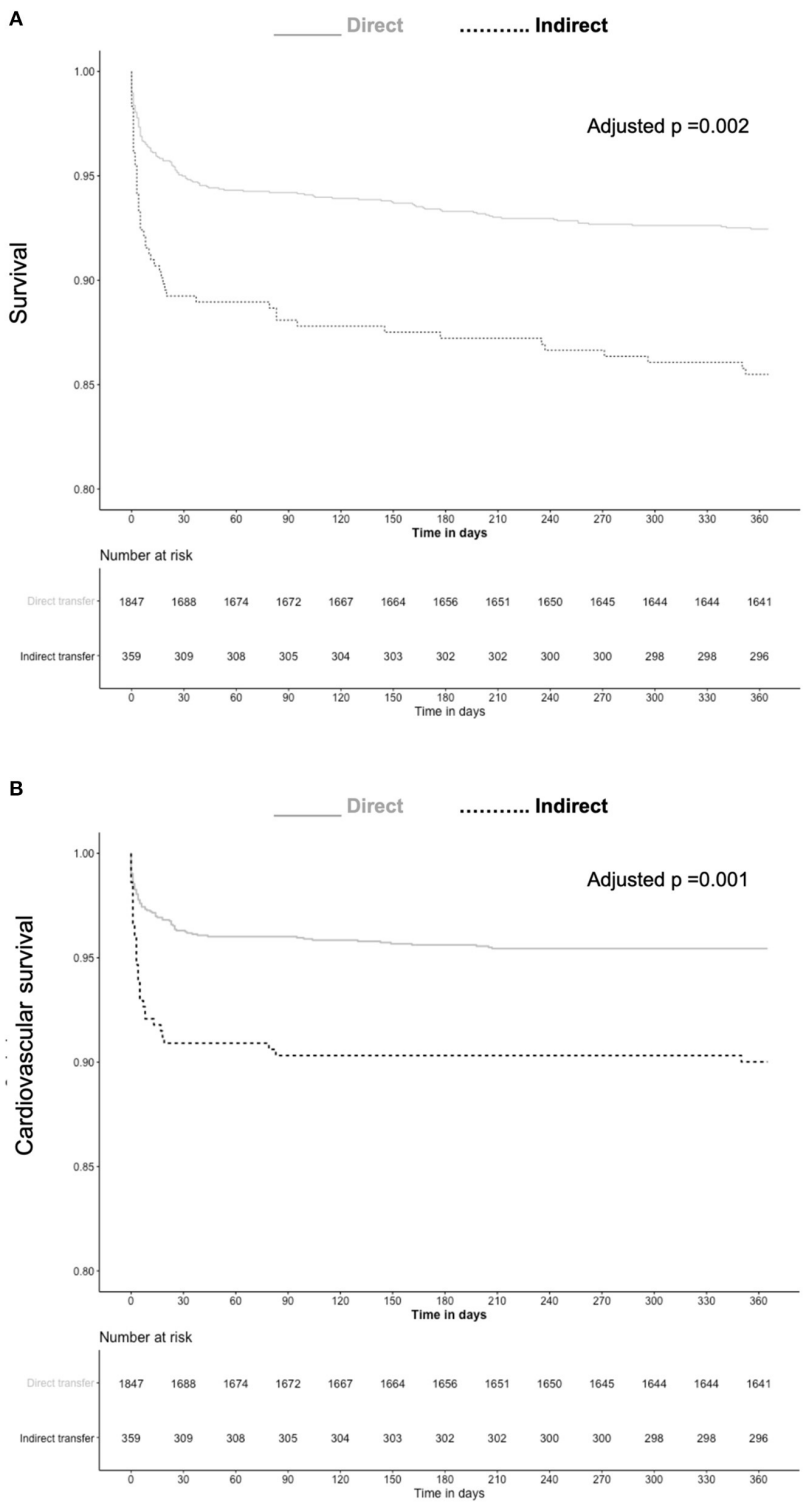
Kaplan-Meier curves for survival **(A)** and cardiovascular survival **(B)** based on direct or indirect admission to catheterization laboratory.

At 1-year follow-up 188 (9%) were reported dead, 51 (14.6%) in the indirect and 137 (7.7%) in the direct admission groups, respectively (adjusted-HR 1.73, 95% CI 1.22–2.45, *p* = 0.002). Cardiovascular death was reported in 35 (10%) and 83 (4.7%) patients in the indirect and direct admission groups, respectively (adjusted-HR 2.01, 95% CI 1.31–3.07, *p* = 0.0003). Figure 2 depicts survival and cardiovascular survival Kaplan-Meier curves. Other 1-year end points were similarly distributed between the groups. At 1-year follow-up 96 and 53% of patients remained on aspirin and a P2Y12 inhibitor, respectively. Such rates as well as the distribution of different P2Y12 inhibitors were similar between the 2 groups suggesting comparable adherence to treatment.

As shown in Table 3, the relationship between the group and, both 1-year mortality and cardiovascular mortality remained significant in models including separately pre-CCL patient (model 1) or intervention characteristics (model 2) both including FMC to balloon time. In model 3 gathering models 1 and 2, and model 4 including in-hospital variables on top of model 3, indirect admission was no more significantly associated with mortality. In the model 4, age, diabetes, Killip class ≥2, FMC to balloon time, pre-CCL P2Y12 inhibitor and anticoagulation, transradial access and successful PCI were independently associated with mortality. All such variables except diabetes, were also independently associated with cardiovascular mortality.

**Table 3:** Variables significantly associated with 1-year mortality and cardiovascular mortality.

	**Model 1**		**Model 2**		**Model 3**		**Model 4**	
	**HR (95% CI)**	***P* value**	**HR (95% CI)**	***P* value**	**HR (95% CI)**	***P* value**	**HR (95% CI)**	***P* value**
**Mortality**								
Indirect transfer	1.50 (1.08–2.08)	0.02	1.74 (1.20–2.51)	0.003	1.26 (0.86–1.84)	0.2	1.23 (0.83–1.81)	0.3
Age per year	1.06 (1.05–1.08)	<0.0001	–	0.002	1.07 (1.05–1.08)	<0.0001	1.06 (1.05–1.08)	<0.0001
Diabetes	1.88 (1.34–2.64)	0.0003	–	<0.0001	1.88 (1.34–2.65)	0.0003	1.99 (1.41–2.81)	0.0001
Killip class ≥2	7.54 (5.62–10.10)	<0.0001	–	0.001	6.41 (4.73–8.69)	<0.0001	5.81 (4.23–7.98)	<0.0001
FMC to balloon per 10'	–		1.008 (1.003–1.013)		1.007 (1.001–1.01)	0.02	1.007 (1.001–1.01)	0.03
Pre-CCL P2Y12 inhibitor	–		0.28 (0.18–0.42)		0.44 (0.29–0.67)	0.0002	0.55 (0.35–0.86)	0.009
Pre-CCL anticoagulation	–		0.53 (0.37–0.77)		0.60 (0.41–0.88)	0.01	0.66 (0.44–1.01)	0.05
Transradial access	–		–		–		0.66 (0.46–0.94)	0.02
Successful PCI	–		–		–		0.25 (0.16–0.40)	<0.0001
**Cardiovascular mortality**								
Indirect transfer	1.72 (1.15–2.57)	0.009	1.98 (1.26–3.12)	0.003	1.42 (0.90–2.27)	0.1	1.39 (0.86–2.23)	0.2
Age per year	1.06 (1.04–1.08)	<0.0001	–	0.07	1.07 (1.05–1.08)	<0.0001	1.06 (1.04–1.08)	<0.0001
Killip class ≥2	9.51 (6.49–13.93)	<0.0001	–	<0.0001	8.09 (5.44–11.99)	<0.0001	7.07 (4.68–10.68)	<0.0001
FMC to balloon per 10'	–		1.006 (1–1.01)	0.0009	1.005 (0.997–1.01)	0.2	1.004 (0.996–1.01)	0.3
Pre-CCL P2Y12 inhibitor	–		0.25 (0.15–0.40)		0.39 (0.24–0.65)	0.0003	0.49 (0.29–0.84)	0.01
Pre-CCL anticoagulation	–		0.46 (0.29–0.73)		0.50 (0.31–0.0.79)	0.003	0.56 (0.33–0.93)	0.03
Successful PCI	–		–		–		0.21 (0.12–0.35)	<0.0001

*FMC, first medical contact; CCL, cardiac catheterization laboratory; PCI, percutaneous coronary intervention*.

*Models were adjusted on FMC to balloon time and covariables unequally distributed between groups (p < 0.1) at different timepoints: Model 1 included pre-FMC variables: age, gender, diabetes, hypertension, current smoking, Killip class≥ 2, past histories of myocardial infarction and PCI; Model 2 included FMC vraiables: national EMS number call, characteristics of the FMC and, pre-CCL aspirin, P2Y12 inhibitor and intravenous anticoagulation administration; Model 3 included all variables in models 1 and 2; Model 4 included all variables in model 3 and in-hospital variables: transradial access, successful PCI and, per procedure P2Y12 inhibitor, 2B3A inhibitor and intravenous anticoagulation*.

Sensitivity analyses (Supplementary Table 1) showed consistent results.

### Determinants of Indirect Admission

As summerized in Table 4, age (OR 1.02 per year, 95% CI 1.003–1.03, *p* = 0.01), paramedics-EMS (OR 5.94 vs. MD-EMS, 95% CI 3.89–9.01, *p* < 0.0001) and private MD (OR 3.41 vs. MD-EMS, 95% CI 1.86–6.21, *p* < 0.0001) were determinants of indirect admission independent of FMC-to-balloon time and other co-variables.

**Table 4:** Independent correlates of indirect admission to catheterization laboratory.

	**OR (95% CI)**	**Wald *p value***
FMC to balloon per 10′	1.08 (1.06–1.09)	<0.0001
Age per year	1.02 (1.003–1.03)	0.01
Paramedics-EMS vs. MD-EMS	5.94 (3.89–9.01)	<0.0001
Private MD vs. MD-EMS	3.41 (1.86–6.21)	<0.0001

*FMC, first medical contact; MD, medical doctor; EMS, emergency medical services*.

*The model included FMC to balloon time, type of FMC (MD EMS, Paramedics EMS, Private MD, PCI-capable hospital, non-PCI-capable hospital) and all pre-FMC characteristics unequally distributed between groups with a p < 0.1 as reported in Table 1*.

### Pre-CCL Pathways

**Figure 3:**
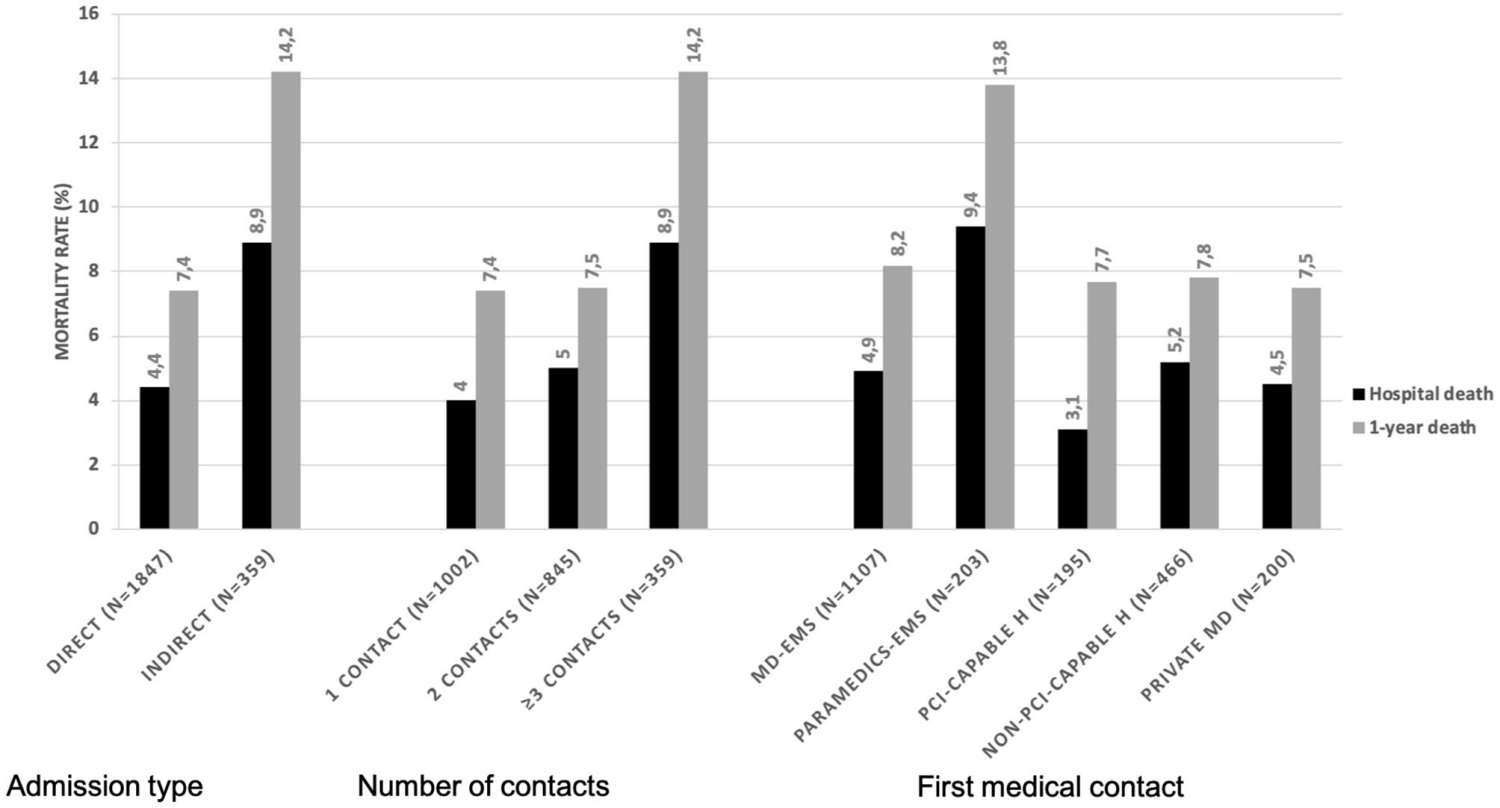
In-hospital and 1-year mortality rates based on different pathways to catheterization laboratory. PCI, percutaneous coronary intervention; EMS, emergency medical services; ED, emergency department; MD, medical doctor; H, hospital.

The Supplementary Table 2 and Figure 3 depict the analysis based on different pre-CCL pathways and their comparison to the MD-EMS group considered as the reference. The analysis adjusted on FMC to balloon time showed that compared to the MD-EMS group, there was an increased risk of in-hospital (adjusted-OR 1.94, 95% CI 1.10–3.31, *p* = 0.02) and 1 year mortality (adjusted-HR 1.62, 95% CI 1.05–2.48, p = 0.02) and cardiovascular mortality (adjusted OR 2.12, 95% CI 1.11–3.85, *p* = 0.02 and adjusted HR 1.85, 95% CI 1.08–3.15, *p* = 0.02) associated with the paramedic-EMS group.

## Discussion

Our study shows that indirect admission of patients to CCL, as defined by guidelines, is associated with more than twice higher risks of both in-hospital and 1-year mortality and cardiovascular mortality. Although indirect admission was associated with increased system delays, its relationship with mortality remained independent of FMC to balloon and total ischemic times. Indirect admission is associated with higher risk features at the time of FMC (older age, female gender, Killip class ≥2, diabetes, hypertension, later presentation), different FMC characteristics (lower rates of national EMS number call, higher rates of paramedics-EMS and self-presentation to ED or to a private MD), pre-CCL medication (lower rates of newer P2Y12 inhibitors and anticoagulation) and in-hospital and procedure characteristics (higher rates of GP2B3A inhibitors and anticoagulation and, lower rates of transradial approach and successful PCI). We identified older age, paramedics-EMS and private MD-FMCs as independent determinants of indirect admission. Finally, paramedics-EMS pathway was associated with higher risks of in-hospital and 1 year mortality as compared to MD-EMS.

System delays are associated with increased mortality and heart failure in the setting of primary PCI for STEMI (2, 13, 14). Based on guidelines, STEMI patients should be transferred directly to the CCL bypassing ED and intensive care units in order to shorten the FMC to balloon times (1, 6). Our study shows that although indirect admission is associated with longer FMC to balloon times, the relationship between indirect admission and mortality remains independent of FMC to balloon time. Such impact of indirect admission on mortality may be explained by several findings in our study. Pre-FMC variables such as older age, female gender, diabetes, hypertension and clinical signs of heart failure (killip class ≥2 and cardiogenic shock), known correlates of mortality were more frequently encountered in the indirect admission group (15–17). Elderly, female and diabetic patients are also more often late presenters, have delayed management and longer total ischemic time and are more likely to have STEMI complicated by heart failure and subsequent mortality (16–18). Concordantly our study shows longer symptom to FMC and total ischemic times in the indirect admission group. However as shown on our multivariable analyses, patient characteristics and longer total ischemic times are not the single explanation to the impact of indirect admission on mortality.

Among FMC-related variables, the pre-CCL medication was different between the groups with higher rates of pretreatment with clopidogrel as compared to newer P2Y12 inhibitors and lower rates of intravenous anticoagulation in those indirectly admitted. Treatment by intravenous anticoagulants and more potent P2Y12 inhibitors rather than clopidogrel is recommended, in the setting of primary PCI based on strong evidence (1). Although the benefit of a pre-CCL administration of ticagrelor as compared to its administration in the CCL on clinical outcome-except in terms of stent thrombosis- or coronary artery flow was not demonstrated in a randomized controlled trial (19), several metanalyses show a benefit of more potent P2Y12 inhibitors over clopidogrel and the benefit of Pre-procedure over per-procedure P2Y12 inhibitors in the setting of STEMI both on clinical endpoints and the quality of coronary flow restoration (20–22). Such discrepancy may be related to the very short time separating the two groups in the latter randomized trial −30′-while in real life, system delays are much longer and allow the P2Y12 inhibitors to be effective at the time of PCI. As a matter of fact a sub-analysis of the above-mentioned trial showed that ticagrelor Pre-treatment was associated with improved reperfusion in those with long transfer delays (23). Pre-CCL anticoagulation has also been reported to be associated with improved coronary flow at the time of PCI (24) especially in those with long transfer delays (23) although an effect on clinical endpoints has not been demonstrated to date. In our study both pre-CCL P2Y12 inhibitor use and anticoagulation were associated with lower rates of mortality at 1 year independent of other covariables. The suboptimal pre-CCL antithrombotic management may expose to the risk of higher thrombus burden at the time of PCI, over-exposure to GP2B3A inhibitors in routine or bail-out situations and suboptimal myocardial reperfusion. The impact of pre-CCL administration of such drugs on coronary flow, a correlate of mortality (25), is supported by our findings of lower rates of GP2B3A inhibition and supplementary anticoagulation as well as the higher rates of successful PCI in those directly admitted to the CCL and may be another explanation of the relationship between the groups and mortality.

Another explanation to the differences in pre-CCL management is the diversity of FMCs and pathways. Unlike other FMCs, private MDs and paramedics-EMS, although capable of activating the CCL, did not administer such medication and systematically required another contact. The latter types of FMC, independently associated with indirect admission in our study, are also associated with longer FMC to balloon times and, no or delayed administration of P2Y12 inhibitors and/or intravenous anticoagulation. A poor impact of presentation to a private-MD vs. EMS on system delays and outcome has been previously reported (26).

Our analysis showed that age, paramedics-EMS and private-MD FMCs were associated with indirect admission independent of FMC to balloon time and other pre-FMC variables. The French EMS targeting STEMI patients are based on a dedicated national number and an MD on board organization. However, MD-EMS were the FMC in only half of patients. In almost 10% of patients the FMC was a paramedics-EMS, and mortality rates were almost twice higher in such patients compared to those managed by MD-EMS. Higher patient risk profile (female gender and Killip class >1) and the lack of medical expertise in the paramedics-EMS potentially leading to delayed diagnosis, patient misorientation and suboptimal management may explain such finding. It is likely that in an emergency care organization based on MD-EMS, paramedic-EMS, associated with indirect admission and increased risk of mortality should be avoided. Another 10% of patients self-presented to their private MD or a non-PCI facility ED. Interestingly, compared to MD-EMS, system delays were significantly higher in all such patients but mortality rates were not higher. Such finding may be due, to a selection bias, less severe status being more likely to lead to self-presentation than EMS call.

Direct admission to CCL was also associated with higher rates of transradial approach and successful PCI both known correlates of decreased mortality in the setting of STEMI (15, 27). Although a relationship between indirect admission and lower rates of transradial approach has previously been reported (8), in absence of a plausible mechanistic explanation such finding may be considered as random. Finally, as mentioned above, the relationship between indirect admission and PCI success may be related to more optimal upfront antithrombotic therapy.

### Limitations

Our study is a retrospective analysis of a prospective registry and may have several limitations inherent to such design. The impact of several known or unknown variables may have been underestimated or unassessed. The registry focuses on the peri-CCL management and antithrombotic therapy; hence the impact of other evidence-based treatments could not be assessed. Furthermore, compliance to evidence-based medication impacting outcomes was not assessed in our study. However similar 1-year rates of antiplatelet therapy suggest comparable adherence to treatment between groups. The findings are specific to the STEMI networks based on MD-EMS as a reference in mixt rural/urban regions and may not apply to other types of organizations. However, none of the latter limitations is likely to impact the main finding of the study. Finally, the multiple analyses based on different FMCs and pathways, should be considered as only exploratory.

## Conclusions

Our study shows that indirect admission to the CCL for primary PCI in the setting of STEMI is associated with in-hospital and 1-year mortality and cardiovascular mortality independent of system delays. Indirect admission to CCL is not only associated with longer FMC to balloon times but also, with higher rates of high-risk patients, suboptimal pre-CCL antithrombotic therapy and lower rates of successful PCI. Among all assessed variables, older age, Paramedics-EMS and self-presentation to a private MD as FMCs were independently associated with indirect admission to CCL. Such findings in a system based on MD-EMS for the management of STEMI, underscores the potential pitfalls of such organization. Our study, highlights the need for population education especially targeting elderly patients and EMS dispatching and staff training in order to improve pre-CCL management and promote direct admission to CCL. Direct admission and optimal pre-CCL management and not only reduced system delays should be considered as STEMI network quality of care measures.

## Data Availability Statement

The data analyzed in this study is subject to the following licenses/restrictions: The data underlying this article were provided by FRANCE-PCI registry. Data will be shared on request to the corresponding author with permission of the FRANCE-PCI registry steering committee. Requests to access these datasets should be directed to FB, beygui-f@chu-caen.r.

## Ethics Statement

The studies involving human participants were reviewed and approved by Commission National Informatique et Libertés-CNIL-, 3 Place de Fontenoy-TSA 80715-75334 PARIS CEDEX 07. The Ethics Committee waived the requirement of written informed consent for participation.

## Author Contributions

FB, RK, and GR contributed to conception and design of the study. GR organized the database. FB performed the statistical analysis and wrote the first draft of the manuscript. All authors contributed to manuscript revision, read, and approved the submitted version.

## Funding

The France-PCI registry's management is funded by the French Regional Health Agencies.

## Conflict of Interest

The authors declare that the research was conducted in the absence of any commercial or financial relationships that could be construed as a potential conflict of interest.

## Publisher's Note

All claims expressed in this article are solely those of the authors and do not necessarily represent those of their affiliated organizations, or those of the publisher, the editors and the reviewers. Any product that may be evaluated in this article, or claim that may be made by its manufacturer, is not guaranteed or endorsed by the publisher.
