# Supplementary material for: Indirect Transfer to Catheterization Laboratory for ST Elevation Myocardial Infarction Is Associated With Mortality Independent of System Delays: Insights From the France-PCI Registry
Source: Front Cardiovasc Med. 2022 Mar 11;9:793067. doi: 10.3389/fcvm.2022.793067 (PMC8962625; doi:10.3389/fcvm.2022.793067)
Supplement: Supplementary file 1 [file Data_Sheet_1.docx]

Supplementary table 1. Sensitivity analyses of the relationship between indirect admission to catheterization laboratory and 1-year mortality

|  | Model 1 | | | Model 2 | | | Model 3 | | | Model 4 | | |
| --- | --- | --- | --- | --- | --- | --- | --- | --- | --- | --- | --- | --- |
|  | HR (95% CI) | P value | HR (95% CI) | | P value | HR (95% CI) | | P value | HR (95% CI) | | P value |  |
| **Analysis 1** |  |  |  | |  |  | |  |  | |  |  |
| Mortality | 1.24 (1.03-1.49) | 0.03 | 1.36 (1.07-1.72) | | 0.01 | 1.14 (0.89-1.46) | | 0.3 | 1.12 (0.87-1.43) | | 0.4 |  |
| Cardiovascular mortality | 1.40 (1.11-1.76) | 0.005 | 1.52 (1.13-2.05) | | 0.006 | 1.26 (0.93-1.71) | | 0.1 | 1.22 (0.90-1.66) | | 0.2 |  |
|  |  |  |  | |  |  | |  |  | |  |  |
| **Analysis 2** |  |  |  | |  |  | |  |  | |  |  |
| Mortality | 1.50 (1.03-1.49) | 0.02 | 1.78 (1.08-2.08) | | 0.003 | 1.25 (0.85-1.83) | | 0.3 | 1.22 (0.83-1.80) | | 0.4 |  |
| Cardiovascular mortality | 1.72 (1.15-2.57) | 0.009 | 1.98 (1.26-3.11) | | 0.003 | 1.43 (0.90-2.23) | | 0.1 | 1.39 (0.86-2.23) | | 0.2 |  |

**Analysis 1**: replacement of the binary variable direct versus indirect admission by the number of contacts (1, 2 , ≥3) prior to catheterization laboratory in the models; Analysis 2: replacement of the variable first medical contact to balloon time by the total ischemic time in the models. **Analysis 2**: replacement of the variable first medical contact to balloon time by the total ischemic time in the models

Models were adjusted on FMC to balloon time and covariables unequally distributed between groups (p<0.1) at different timepoints: Model 1 included pre-FMC variables: age, gender, diabetes, hypertension, current smoking, Killip class≥ 2, past histories of myocardial infarction and PCI; Model 2 included FMC vraiables: national EMS number call, characteristics of the FMC and, pre-CCL aspirin, P2Y12 inhibitor and intravenous anticoagulation administration; Model 3 included all variables in models 1 and 2; Model 4 included all variables in model 3 and in-hospital variables: transradial access, successful PCI and, per procedure P2Y12 inhibitor, 2B3A inhibitor and intravenous anticoagulation

FMC: first medical contact, CCL: cardiac catheterization laboratory, PCI: percutaneous coronary intervention

Supplementary table 2. Characteristics based on first medical contact pathways

|  | MD-EMS (reference) | ED/ward of Non-PCI hospital | p | ED/ward of PCI-hospital | p | Private MD | p | Paramedics-EMS | p |
| --- | --- | --- | --- | --- | --- | --- | --- | --- | --- |
| Age, years  Gender female  BMI, Kg.m^-2^  Killip class > 1 | 62.7±13.4  253(22.9)  26.6±4.2  166(15) | 61.9±14.8  136(27.2)  26.8±4.7  76(15.2) | NS  0.06  NS  NS | 62±13.7  41(21)  26.7±4.2  29(14.9) | NS  NS  NS  NS | 63.9±12.7  51(25.5)  27.1±4.9  22(11) | NS  NS  NS  NS | 64±15.5  66(32.5)  26.1±4.3  42(20.7) | NS  0.003  NS  0.04 |
| **Past history**  Diabetes  Hyperlipemia  Current smoking  Hypertension  PCI  Myocardial infarction  Stroke  PAD  Coronary bypass | 139(12.6)  433(39.1)  429(38.8)  432(39)  152(13.7)  99(8.9)  32(2.9)  42(3.8)  17(1.5) | 72(14.4)  179(35.7)  198(39.5)  206(41.1)  40(8)  33(6.6)  13(2.6)  13(2.6)  4(0.8) | NS  NS  NS  NS  0.001  NS  NS  NS  NS | 27(13.9)  61(31.3)  75(38.5)  78(40)  53(27.2)  26(13.3)  2(1)  8(4.1)  4(2) | NS  0.04  NS  NS  <0.0001  0.06  NS  NS  NS | 33(16.5)  71(35.5)  61(30.5)  85(42.5)  12(6)  10(5)  2(1)  4(2)  2(1) | NS  NS  0.03  NS  0.003  0.07  NS  NS  NS | 24(11.8)  70(34.5)  75(37)  81(39.9)  20(9.9)  9(4.4)  4(2)  5(2.5)  3(1.5) | NS  NS  NS  NS  NS  0.04  NS  NS  NS |
| **Pre-catheterization data**  EMS number call  Times, min  Symptom to FMC  FMC to door  Door to balloon  FMC to balloon  FMC to balloon <120’  FMC to balloon <90’  Total ischemic time  Medication  Aspirin  P2Y12 inhibitors  Clopidogrel  Prasugrel  Ticagrelor  IV anticoagulation  Enoxaparin  UFH  Bivalirudin | 1090(98.5)  131.9±156.2  78.8±64.6  42.1±93  120.9±119.6  779(70.4)  463(42)  252.9±203.4  1060(95.8)  1026(92.7)  101(9.1)  84(7.6)  841(76)  1015(91.7)  453(41)  549(49.6)  13(1.2) | 31(6.2)  226.8±249.5  142.8±119.4  75.5±213.6  218.3±246  109(21.8)  48(10)  445±351.2  481(96)  476(95)  75(15)  23(4.6)  378(75.5)  446(89)  187(37)  251(50.1)  6(1.2) | <0.0001  <0.0001  <0.0001  <0.0001  <0.0001  <0.0001  <0.0001  <0.0001  NS  0.08  0.001  0.03  NS  0.09  NS  NS  NS | 8(4.1)  184.1±264.1  2.5±10.6  78.6±74.4  81.1±74  166(85.1)  146(75)  265.1±271.9  176(90.3)  172(88.2)  29(14.9)  11(5.6)  132(67.7)  156(80)  66(34)  90(46.2)  0(0) | <0.0001  0.001  <0.0001  0.001  0.001  <0.0001  <0.0001  NS  0.002  0.04  0.02  NS  0.02  <0.0001  0.06  NS  NS | 6(3)  315.8±324.2  143.3±127.2  55±76.4  198.3±146.2  47(23.5)  17(9)  514.1±362.2  190(95)  182(91)  22(11)  14(7)  146(73)  175(87.5)  66(33)  106(53)  3(1.5) | <.0001  <.0001  <.0001  NS  <0.0001  <0.0001  <0.0001  <0.0001  NS  NS  NS  NS  NS  0.06  0.04  NS  NS | 110(54.2)  136.1±136.7  81.4±91.8  62±120.1  143.4±153.5  121(59.6)  71(35)  279.5±214.3  191(94.1)  174(85.7)  24(11.8)  3(1.5)  147(72.4)  176(86.7)  73(36)  102(50.3)  0(0) | <0.0001  NS  NS  <0.05  0.07  0.003  0.07  NS  NS  0.001  NS  0.004  NS  0.02  NS  NS  NS |
| **In-hospital data**  Per-procedure medication  Aspirin  P2Y12 inhibitors  Clopidogrel  Prasugrel  Ticagrelor  2B3A inhibitors  IV anticoagulation  Enoxaparin  UFH  Bivalirudin  Transradial approach  Left main disease  Single vessel disease  Successful PCI  Stents per patient  Hospitalization length, days | 203(18.3)  90(8.1)  10(0.9)  7(0.6)  73(6.6)  441(39.8)  549(49.6)  10(0.9)  535(48.3)  6(0.5)  1019(92)  1(0.09)  497(44.9)  1084(97.9)  1.2±0.7  6.7±7.3 | 74(14.8)  33(6.6)  7(1.4)  1(0.2)  25(5)  143(28.5)  269(53.7)  7(1.4)  263(52.5)  2(0.4)  461(92)  3(0.6)  248(49.5)  485(96.8)  1.22±0.8  5.4±4.9 | 0.08  NS  NS  NS  NS  <0.0001  NS  NS  NS  NS  NS  NS  0.09  NS  NS  0.002 | 34(17.4)  17(8.7)  4(2)  1(0.5)  12(6.2)  103(52.8)  111(56.9)  0(0)  111(56.9)  0(0)  166(85.1)  0(0)  98(50.3)  193(99)  1.1±1  6.5±4.9 | NS  NS  NS  NS  NS  0.0008  0.06  NS  0.03  NS  0.002  NS  NS  NS  0.05  NS | 33(16.5)  14(7)  1(0.5)  1(0.5)  12(6)  65(32.5)  100(50)  1(0.5)  98(49)  2(1)  183(91.5)  0(0)  86(43)  194(97)  1.26±0.8  7.7±13.3 | NS  NS  NS  NS  NS  0.05  NS  NS  NS  NS  NS  NS  NS  NS  NS  0.08 | 38(18.7)  20(9.9)  2(1)  1(0.5)  17(8.4)  78(38.4)  112(55.2)  1(0.5)  111(54.7)  0(0)  176(86.7)  0(0)  83(40.9)  195(96)  1.2±0.9  7.5±8.4 | NS  NS  NS  NS  NS  NS  NS  NS  NS  NS  0.01  NS  NS  NS  NS  NS |

FMC: first medical contact; BMI: body mass index; ED: emergency department; PCI: percutaneous coronary intervention, PAD: peripheral arterial disease; EMS: emergency medical services; MD: medical doctor; PCI: percutaneous coronary intervention; MI: myocardial infarction, IV: intra venous, UFH: unfractionated heparin
